# Supplementary material for: Forecasting hospital-level COVID-19 admissions using real-time mobility data
Source: Commun Med (Lond). 2023 Feb 14;3:25. doi: 10.1038/s43856-023-00253-5 (PMC9927044; doi:10.1038/s43856-023-00253-5)
Supplement: Supplementary file 1 — Supplementary Information [file 43856_2023_253_MOESM1_ESM.pdf]

Supplemental Figures

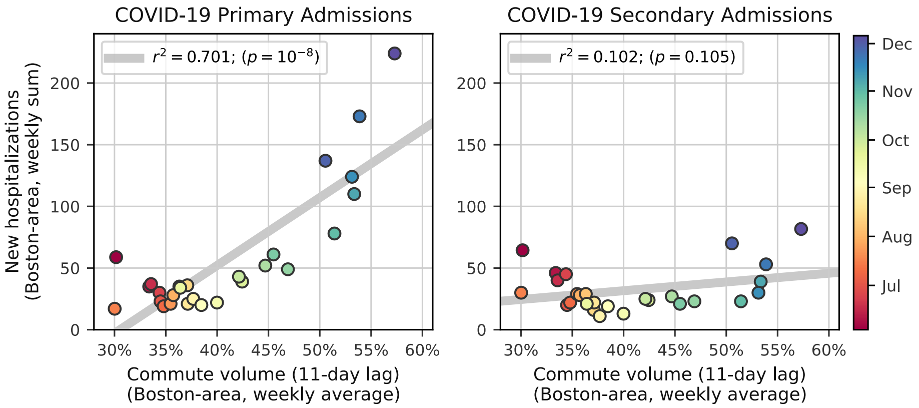

Supplementary Figure 1: Commute volume and hospital admissions. From June to December 2020, commute volume began to increase in the greater Boston area. This coincided with an increase in weekly COVID-19 "primary" hospital admissions (left), though we do not see the same correlations with COVID-19 "secondary" admissions (right).

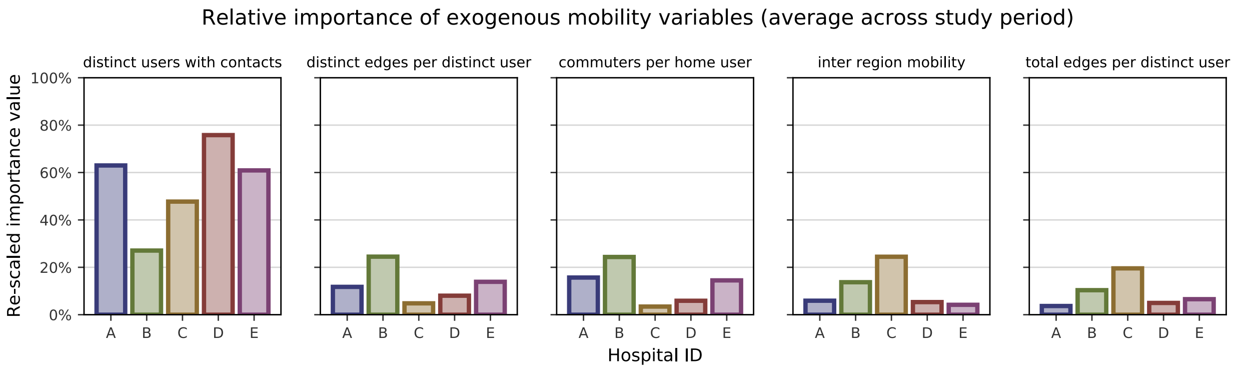

Supplementary Figure 2: Relative importance of exogenous mobility variables. For each of the exogenous mobility variables included in our forecasting models, we can assign a value that corresponds to its relative importance in the overall accuracy of the predictions (for each hospital separately). The scores in the plot above have been rescaled and in order to highlight the relative differences in feature importance within the exogenous mobility variables.

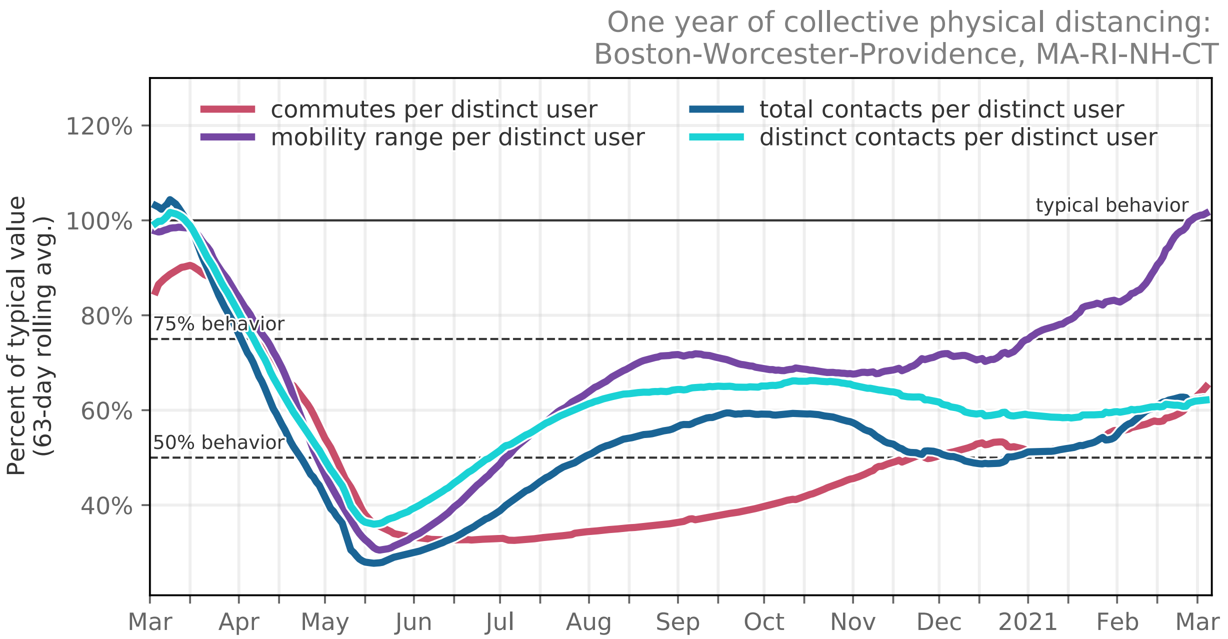

Supplementary Figure 3: Mobility and contact patterns of the greater Boston combined statistical area. (CSA; Boston-Worcester-Providence, MA-RI-NH-CT).

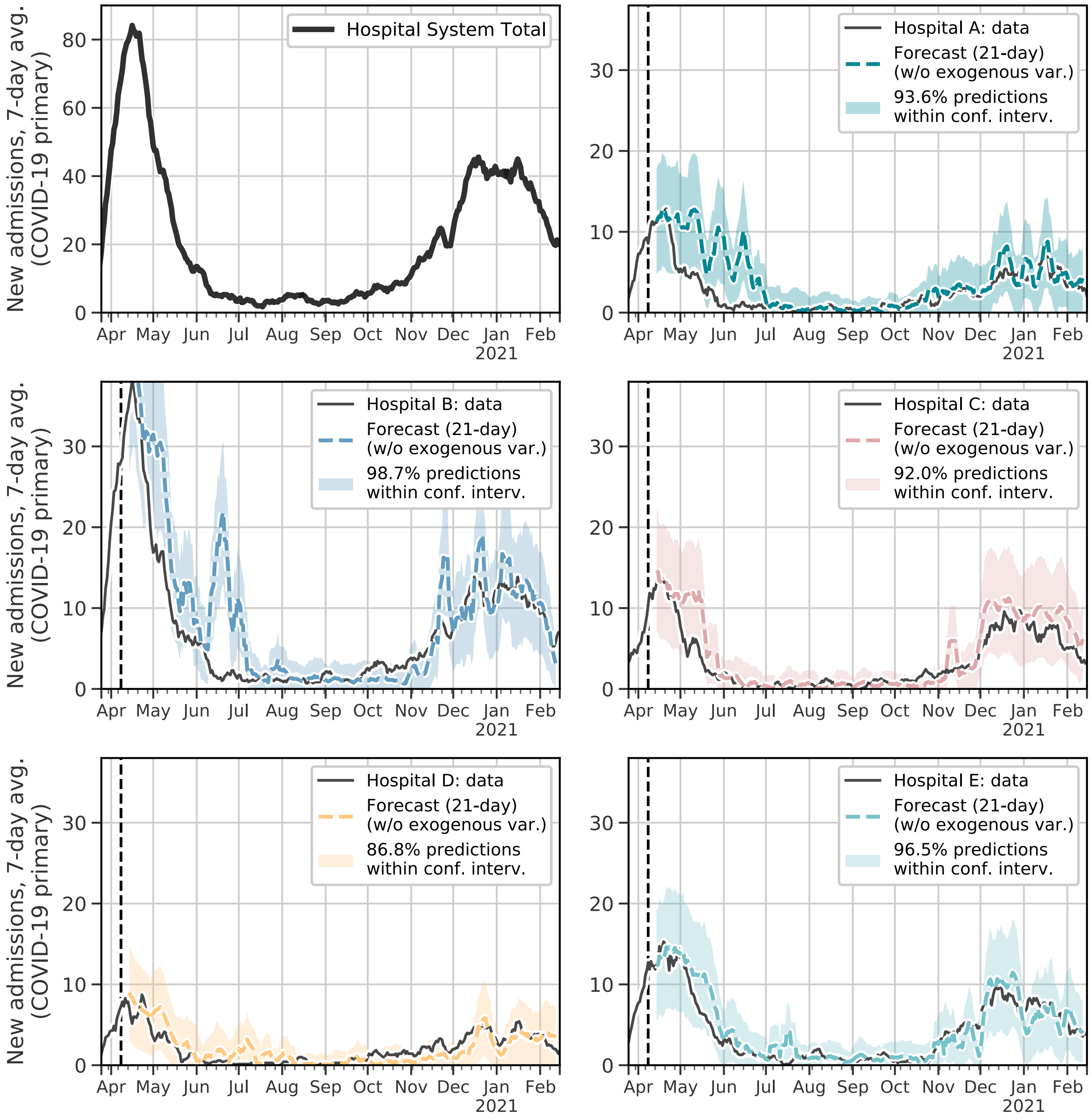

Supplementary Figure 4: 21-day forecast for each hospital without including additional exogenous data. Similar to Fig. 2, the vertical dashed line marks the boundary of boundary of initial training data and future data; visualized at a 7-day rolling average; 95% confidence intervals are shown for each forecast. Hyper-parameters of each model were tuned separately to make sure fair comparisons were made between models.
