# Supplementary material for: Forecasting hospital-level COVID-19 admissions using real-time mobility data
Source: Commun Med (Lond). 2023 Feb 14;3:25. doi: 10.1038/s43856-023-00253-5 (PMC9927044; doi:10.1038/s43856-023-00253-5)
Supplement: Supplementary file 2 — Reporting Summary [file 43856_2023_253_MOESM2_ESM.pdf]

nature portfolio

Corresponding author(s): *Double-blind peer review submissions: write DBPR and your manuscript number here instead of author names.*

Last updated by author(s): *YYYY-MM-DD*

Reporting Summary

Nature Portfolio wishes to improve the reproducibility of the work that we publish. This form provides structure for consistency and transparency in reporting. For further information on Nature Portfolio policies, see our Editorial Policies and the Editorial Policy Checklist.

Statistics

For all statistical analyses, confirm that the following items are present in the figure legend, table legend, main text, or Methods section.

n/a	Confirmed	
☐	☒	The exact sample size (*n*) for each experimental group/condition, given as a discrete number and unit of measurement
☒	☐	A statement on whether measurements were taken from distinct samples or whether the same sample was measured repeatedly
☒	☐	The statistical test(s) used AND whether they are one- or two-sided<br>*Only common tests should be described solely by name; describe more complex techniques in the Methods section.*
☐	☒	A description of all covariates tested
☒	☐	A description of any assumptions or corrections, such as tests of normality and adjustment for multiple comparisons
☒	☐	A full description of the statistical parameters including central tendency (e.g. means) or other basic estimates (e.g. regression coefficient) AND variation (e.g. standard deviation) or associated estimates of uncertainty (e.g. confidence intervals)
☒	☐	For null hypothesis testing, the test statistic (e.g. *F*, *t*, *r*) with confidence intervals, effect sizes, degrees of freedom and *P* value noted<br>*Give P values as exact values whenever suitable.*
☒	☐	For Bayesian analysis, information on the choice of priors and Markov chain Monte Carlo settings
☒	☐	For hierarchical and complex designs, identification of the appropriate level for tests and full reporting of outcomes
☒	☐	Estimates of effect sizes (e.g. Cohen's *d*, Pearson's *r*), indicating how they were calculated

Our web collection on statistics for biologists contains articles on many of the points above.

Software and code

Policy information about availability of computer code

Data collection	This study used three primary data sources: The first is data about COVID-19 admissions from a network of hospitals in Massachusetts. The second is statewide data about daily test positivity for Massachusetts. The third is data from Cuebiq Inc., which shared anonymized, privacy-preserving data about aggregate user mobility. The data underlying this article will be shared on reasonable request to the corresponding author. The Cuebiq Inc. Data for Good initiative is currently known as Spectus and more information can be found at https://spectus.ai/
Data analysis	All analyses were conducted in Python, using the skforecast library. Code underlying the modeling, analysis, and creation of figures is available at https://github.com/jkbren/hospital-covid19-forecasts or archived in the following Zenodo repository: doi: 10.5281/zenodo.7459095.

For manuscripts utilizing custom algorithms or software that are central to the research but not yet described in published literature, software must be made available to editors and reviewers. We strongly encourage code deposition in a community repository (e.g. GitHub). See the Nature Portfolio guidelines for submitting code & software for further information.

Data

Policy information about availability of data

All manuscripts must include a data availability statement. This statement should provide the following information, where applicable:

- Accession codes, unique identifiers, or web links for publicly available datasets
- A description of any restrictions on data availability
- For clinical datasets or third party data, please ensure that the statement adheres to our policy

The data underlying this article will be shared on reasonable request to the corresponding author. The Cuebiq Inc. Data for Good initiative is currently known as Spectus and more information can be found at https://spectus.ai/.

Human research participants

Policy information about studies involving human research participants and Sex and Gender in Research.

Reporting on sex and gender	N/A
Population characteristics	See above.
Recruitment	N/A
Ethics oversight	Because the data sources used in this study were only used in aggregated form, the Institutional Review Board (IRB) determined that the study does not qualify as human subject research and is exempt from IRB approval.

Note that full information on the approval of the study protocol must also be provided in the manuscript.

Field-specific reporting

Please select the one below that is the best fit for your research. If you are not sure, read the appropriate sections before making your selection.

☐ Life sciences ☒ Behavioural & social sciences ☐ Ecological, evolutionary & environmental sciences

For a reference copy of the document with all sections, see nature.com/documents/nr-reporting-summary-flat.pdf

Behavioural & social sciences study design

All studies must disclose on these points even when the disclosure is negative.

Study description	The data are quantitative time series of hospital admissions, human mobility, and COVID-19 test positivity.
Research sample	For the mobility data, we use a subset from a panel of users in the Cuebiq Inc. dataset (via a protocol defined in the Methods section of the manuscript). Hospital admissions data were not sampled, we use all of the COVID-19 admissions at five separate hospitals in this network of Massachusetts hospitals.
Sampling strategy	From a nationwide sample of over 40 million mobile devices, we generate a panel of users with personal areas in the Boston Combined Statistical Area (CSA ID: 148; BostonWorcester-Providence, MA-RI-NH-CT). In order to reduce the noise in user activity (e.g. turnover in device, deleting apps, etc.), we devised strict criteria for inclusion in the panel. We include users in our panel who 1) were active since January 7, 2020, 2) were active for at least 21 days per month between January and June, 2020, 3) generated at least one location ping per hour, on average, and 4) had an average geolocation accuracy of less than 50 meters. This resulted in a panel with over 82,000 mobile devices, selected for their accuracy and stability in the dataset. Lastly, the measures derived from this panel are re-weighted (at the county level) by a statistical correction procedure designed to maximize the representativeness of the panel based on county-level demographic data. For more information on the methods involved in this representativeness correction, see https://www.mobs-lab.org/uploads/6/7/8/7/6787877/covid19mobility_report2.pdf.
Data collection	The mobility data is collected by Cuebiq Inc., and the hospital admissions data is collected and maintained by a large hospital network in Massachusetts.
Timing	The study period ranges during roughly the first year of the COVID-19 pandemic in Massachusetts. We selected this period in particular because this was a period without widespread vaccination, and it was while mobility had not yet returned to pre-pandemic levels.
Data exclusions	N/A
Non-participation	N/A

Randomization	N/A

Reporting for specific materials, systems and methods

We require information from authors about some types of materials, experimental systems and methods used in many studies. Here, indicate whether each material, system or method listed is relevant to your study. If you are not sure if a list item applies to your research, read the appropriate section before selecting a response.

Materials & experimental systems

n/a	Involved in the study
☒	☐ Antibodies
☒	☐ Eukaryotic cell lines
☒	☐ Palaeontology and archaeology
☒	☐ Animals and other organisms
☒	☐ Clinical data
☒	☐ Dual use research of concern

Methods

n/a	Involved in the study
☒	☐ ChIP-seq
☒	☐ Flow cytometry
☒	☐ MRI-based neuroimaging
